# I Am Afraid I Will Not Be Able to Walk, That Is What Worries Me—The Experience of Patients with Knee Osteoarthritis before Total Knee Arthroplasty: A Qualitative Study

**DOI:** 10.3390/jcm13102878

**Published:** 2024-05-13

**Authors:** Umile Giuseppe Longo, Alessandra Corradini, Anna Marchetti, Chiara Di Sarno, Carlotta D’Angelo, Claudia Arias, Maria Grazia De Marinis, Alessandro de Sire, Vincenzo Denaro

**Affiliations:** 1 Fondazione Policlinico Universitario Campus Bio-Medico, 00128 Roma, Italy; a.corradini@policlinicocampus.it (A.C.); v.denaro@policlinicocampus.it (V.D.); 2 Research Unit of Orthopaedic and Trauma Surgery, Department of Medicine and Surgery, Università Campus Bio-Medico di Roma, 00128 Roma, Italy; chiara.disarno@alcampus.it (C.D.S.); carlotta.dangelo@alcampus.it (C.D.); 3 Research Unit Nursing Science, Campus Bio-Medico University, 00128 Rome, Italy; a.marchetti@policlinicocampus.it (A.M.); m.demarinis@unicampus.it (M.G.D.M.); 4 Hospital Nacional Edgardo Rebagliati Martins, Lima 15072, Peru; claudia_arias81@hotmail.com; 5 Physical Medicine and Rehabilitation Unit, Department of Medical and Surgical Sciences, University of Catanzaro “Magna Graecia”, 88100 Catanzaro, Italy; alessandro.desire@unicz.it; 6 Research Center on Musculoskeletal Health, MusculoSkeletalHealth@UMG, University of Catanzaro “Magna Graecia”, 88100 Catanzaro, Italy

**Keywords:** knee osteoarthritis, activity of daily living, qualitative research, pain, knee pain, rehabilitation

## Abstract

**Background:** Knee osteoarthritis is the most prevalent type of osteoarthritis. Patients frequently encounter pain triggered by movement that evolves into impaired joint function. Needing persistent rest or having night-time pain signifies advanced disease. Qualitative research is considered the most effective method for comprehending patients’ needs and contexts. **Methods:** This study employed a qualitative research design, allowing the researchers to acquire insights into the patients’ beliefs and values, and the contextual factors influencing the formation and expression of these beliefs and values. **Results:** A cohort of nine patients awaiting total knee replacement (TKR) surgery was included and they were interviewed until data saturation was achieved. The results of the phenomenological analysis resulted in the identification of three themes: “The existence of pain impedes the capacity to participate in daily life activities”; “TKR induced fears and uncertainties regarding the progression of the disease”; “Severe nighttime pain compromising sleep quality”. **Conclusions:** This study analyzes the experiences of people awaiting TKR surgery, emphasizing the importance of addressing their unique needs to improve preoperative education and rehabilitation. In this way, patients’ recovery during the postoperative phase can be improved.

## 1. Introduction

Osteoarthritis (OA) stands as the prevailing joint pathology in adults worldwide. OA is a persistent joint pathology characterized by the progressive degeneration and erosion of articular cartilage, accompanied by the formation of reactive subchondral bone tissue and joint margins. While it can affect any joint, its main sites of involvement are the spine, hip, knee, hand, and foot, but knee OA (KOA) is the most prevalent type [1]. Radiological evidence of typical KOA signs does not consistently correlate with symptomatic presentation. Epidemiological investigations have unveiled a combination of endogenous and exogenous risk factors contributing to the development of osteoarthritis [2]. KOA is categorized into primary (idiopathic) and secondary forms. Within the complex knee joint structures, the hyaline joint cartilage represents the primary site of detrimental influences leading to osteoarthritis, serving as the initial location where the disease initiates. The diagnostic evaluation of OA comprises patient history, physical examination, imaging, and when necessary, laboratory testing. Physical examination includes inspection, palpation, range of motion tests, and functional assessments (for example, consisting of ligament stability, meniscus tests, gait analysis…). X-ray is used for diagnosis and disease progression assessment, while MRI can demonstrate hyaline cartilage [2].

Patients commonly experience movement-induced pain, initially described as a dull ache, progressing to continuous pain and joint dysfunction. Knee pain worsens during motion but eases at rest, with the need for persistent rest or the presence of night-time pain indicating advanced disease. An additional manifestation is joint stiffness, frequently encountered upon awakening or after extended periods of rest, yet demonstrating improvement with movement [3].

Treatment of KOA can be categorized into non-surgical and surgical approaches. Non-surgical treatment includes changes in lifestyle (e.g., encompassing education, self-management, physical activity, and weight reduction), pharmacological therapy (paracetamol, topical or oral non-steroidal anti-inflammatory drugs, and intra-articular injections of corticosteroids and hyaluronic acid), nutraceuticals, physical therapy, oxygen–ozone therapy, and rehabilitation, all of which might play a key role, particularly in KOA patients in their early stages [4,5,6,7]. In recent years, radiofrequency ablation (CRFA) has emerged as a promising alternative treatment modality for KOA. In contrast to conventional radiofrequency procedures, CRFA employs advanced cooling technology to target and modulate sensory nerves responsible for pain signal transmission [8].

Surgical options are reserved for end-stage KOA, with total knee replacement (TKR) along with rehabilitation being the most effective procedure. Exercise aims to alleviate pain, enhance overall mobility, and improve joint function, with intensive exercise further targeting knee joint muscle strengthening [9,10].

TKR is more frequently performed on the aging population, which comprises several risk factors associated with the development of depression. These risk factors include social isolation, lower socioeconomic status, general medical conditions, uncontrolled pain, and reduced sleep quality [9,11].

The objective of this study is to explore patients’ experiences during the pre-admission phase for TKR.

Currently, there is a paucity of studies that have examined the qualitative aspects of patient experiences following TKR. While some studies indicate that patients report enhanced quality of life, reduced pain, and improved mobility after TKR, others highlight issues such as dissatisfaction with prosthesis function and esthetic appearance, and limited range of motion [12]. There is a need for further research to gain a comprehensive understanding of patients’ perspectives on TKR and to establish a standardized approach for evaluating patient satisfaction [13,14].

Qualitative research is considered the most effective method for comprehending patients’ needs and contexts, which could enhance therapeutic strategies and their assessment. Understanding patients’ emotions and feelings before surgery can aid in identifying and addressing potential challenges earlier.

## 2. Materials and Methods

### 2.1. Design

The aim of qualitative research is to explore, describe, and interpret the human experience, with interviews being the primary method used for generating qualitative data [15].

This study employed a qualitative research design, allowing the researchers to acquire insights into the patients’ beliefs and values, and the contextual factors influencing the formation and expression of these beliefs and values [16].

Interviews represent a commonly used data collection method in nursing research.

These interviews were performed employing a phenomenological approach, which facilitates the exploration and comprehension of the patients’ inner world, encompassing emotions, perceptions, experiences, and perspectives [17].

The descriptions are comprehensive and articulated in a language that is readily understandable to individuals experiencing the phenomenon.

Giorgi’s analytical method strives to reveal the essential nature of the phenomenon as perceived by individuals, achieved through the identification and analysis of critical themes [18].

This method encompasses five steps:

- Gathering verbal data (this initial phase involves the collection of spoken or verbal information relevant to the subject under investigation).
- Thoroughly reviewing the data (subsequently, a comprehensive examination of the gathered data is undertaken, ensuring a meticulous review of the content and context).
- Breaking down the data into discrete elements (the acquired data are then systematically dissected into distinct and separate elements, facilitating a granular analysis of the components).
- Structuring and presenting the data from a disciplinary standpoint (following dissection, the data are organized and presented in a structured manner, aligning with the principles and frameworks pertinent to the discipline in question).
- Synthesizing or consolidating the data to reveal the underlying pattern of the phenomenon (finally, the synthesized or consolidated form of the data is generated, aiming to unveil the inherent patterns and trends associated with the phenomenon under investigation; this step involves a holistic integration of the dissected elements to provide a comprehensive understanding) [19].

### 2.2. Participants

The data were collected using unstructured interviews, with interview scheduling derived from the pre-admission list for surgical procedures. The inclusion criteria comprised adult patients undergoing pre-admission for TKR due to KOA, who provided informed consent to participate in this study. The exclusion criteria encompassed patients who did not provide consent for participation and data usage, individuals below 18 years of age, those lacking proficiency in the Italian language, and individuals with cognitive deficits impeding their ability to describe their experiences. All participants were provided with comprehensive explanations concerning the research objectives and interview procedures. They provided their informed consent to participate and were interviewed until adequate information was attained. The conclusion of the interviews was reached when no further significant information emerged from subsequent participants.

### 2.3. Setting and Data Collection

Between July 2023 and August 2023, qualitative data were collected through interviews conducted at the Orthopedic and Traumatological Surgery Department of a university hospital in Rome, Italy. Participants were selected from the pre-admission list for surgery and subsequently interviewed until data saturation. We arrived at saturation through the use of a grid in Excel, entering codes according to topics; in the columns, we entered the patient data and cross-referenced the data to see if and how much the codes were repeated. Data saturation was calculated by seeing if, at least in the last interview, I had no new codes. In the final interviews, there were no new arguments, so saturation of the data was reached. The interviews, lasting between 5 and 15 min, were digitally recorded and transcribed verbatim. Before commencing the interviews, the research team engaged in discussions with the participants regarding the central themes of the study, encouraging them to respond narratively to the questions. Subsequently, the participants provided informed consent. The questions were carefully crafted to afford respondents the flexibility to shape the conversation’s trajectory while ensuring the inclusion of essential topics for exploration. The subsequent inquiries were posed: Can you tell me about your health problem? What changes (physical, psychological, emotional, etc.) did it bring about in your life? What are your fears (emotions/thoughts) about the future of your illness? How do you sleep? How is the quality of sleep? Would you like to add more?

### 2.4. Data Analysis

The data analysis was conducted employing Giorgi’s descriptive phenomenological approach. In the initial stage, the researchers acquired verbal data through audio recordings of the interviews. Subsequently, the transcriptions of the interviews were generated verbatim daily to ensure the precision and reliability of the gathered data. The transcription process retained the participants’ exact expressions. In the subsequent phase of data analysis, the researchers meticulously reviewed the transcriptions with an unbiased perspective to develop complete comprehension of the gathered information. During the third step, the transcriptions were partitioned into smaller segments, and each segment underwent a line-by-line re-examination to discern the “units of meaning”, which represent discrete and meaningful elements within the descriptions. In the fourth step, every identified unit of meaning was subjected to analysis and reorganization to augment its disciplinary significance and articulate it in terms pertinent to the clinical context. Finally, in the concluding step, akin units of meaning were aggregated and amalgamated into more extensive subcategories to synthesize the data’s overall significance. During the abstraction phase, subcategories exhibiting conceptual and semantic resemblances were further grouped into generic categories. These categories were formulated to encapsulate the fundamental essence of the data, and their inter-relationships were elucidated by identifying themes and sub-themes, thus making the connections among them explicit. A senior researcher proficient in qualitative research supervised all stages of the analysis. The research team participated in discussions to reconcile any disparities, ultimately reaching a consensus.

### 2.5. Trustworthiness

To uphold the validity and reliability of the findings, this study employed the Lincoln and Guba criteria, which assess the trustworthiness of qualitative research concerning aspects such as credibility, reliability, confirmability, and transferability. To augment credibility, the research team extended the sampling and data collection process until data saturation was reached. The utilization of the triangulation technique, involving multiple researchers in the analysis process, ensured reliability. This approach offers diverse viewpoints, validating the findings and enriching the study’s understanding of the phenomenon, resulting in multiple conclusions. Confirmability was achieved through audit trails, wherein two independent researchers scrutinized the survey process to ascertain that the results aligned with the data. Transferability was substantiated by providing a detailed account of the socio-demographic characteristics of the participants and a comprehensive, precise description of the methodology, research process, and findings.

### 2.6. Ethical Considerations

This study was conducted according to the Declaration of Helsinki and was ap- proved by the Institutional Review Board of Campus Bio-Medico University of Rome (COSMO study, protocol number: 78/18 OSS ComEt CBM, 16/10/18). On the pre-admission day, eligible patients were contacted via telephone and given verbal information regarding the study’s purpose, the interview method, and the need for digital audio recording. Before commencing the interview, all participants provided informed consent by signing a consent form, indicating their willingness to participate in the study. Anonymity was safeguarded using identification codes during the transcription of the interviews and throughout the data analysis process, thereby preventing any individual participant from being identifiable in the study report. Participants were duly informed of their right to withdraw from the study at any point in time.

## 3. Results

The investigation incorporated a cohort of nine patients awaiting TKR surgery. The cohort consisted of eight females and one male, with a mean age of 75 years (70–84 years). All persons without comorbidity of a psychological nature were recruited. Most patients lived within their own household (at least with a cohabitant), while a couple lived alone but with supportive families present (Table 1).

Three main themes are deducted from this phenomenological analysis. The first theme is “The existence of pain impedes the capacity to participate in daily life activities”; the second theme is “TKR induced fears and uncertainties regarding the progression of the disease”; the third theme is “Severe nighttime pain compromising sleep quality”.

*First theme: “The existence of pain impedes the capacity to participate in daily life activities”.*

KOA induces a level of disability in patients, impeding their ability not only to carry out routine daily activities but also to participate in the sports they used to do.

*“I had to change my whole lifestyle. I no longer did sports as I liked. I did swimming, cycling, football. I also liked to go dancing…”*

(M, 72 years)

*“I used to do a lot of sports, I used to swim. I also used to do walking and I don’t do that anymore. The only sport I do now is postural gymnastics and I started walking again two months ago.”*

(F, 70 years)

OA may lead to reduced mobility and substantial physical impairment, impacting an individual’s autonomy in executing daily activities such as walking, climbing stairs, bathing, and transitioning from a seated position.

*“I used to be autonomous but since the problem arose, I am no longer, I always need the stick and my children.”*

(F, 70 years)

*“I have completely lost my autonomy, I can no longer do what I used to do, I even find it difficult to go to the toilet if I am not accompanied.”*

(F, 76 years)

*“…slows me down both in walking and in going up and down stairs leading me to use a stick or something to lean on.”*

(F, 75 years)

*Second theme: TKR induced fears and uncertainties regarding the progression of the disease.*

From the qualitative study conducted, it emerges that patients awaiting total knee arthroplasty develop a sense of fear and uncertainty associated with the lack of knowledge about the postoperative period. It is highly common to observe in patients a fear of being unable to resume walking. Patients frequently express a strong desire to return to pain-free walking, and this becomes a predominant hope for their postoperative period.

*“My fear is that I will suffer and not be able to walk properly.”*

(M, 72 years)

*“The main fear is that I may walk badly…”*

(F, 70 years)

*“I’m afraid I won’t be able to walk, that’s what worries me.”*

(F, 84 years)

*“I am afraid of not being able to walk properly and of having pains”.*

(F, 73 years)

There is apprehension regarding the ability to regain the desired level of ambulation, and patients express hope for a successful surgery that would restore the autonomy required for engaging in activities of daily living.

*“My fear is that I won’t resolve post-surgery, but I hope to get back to doing things independently…”.*

(F, 70 years)

*“I hope the surgery will restore my autonomy”.*

(F, 76 years)

*Third theme: “Severe nighttime pain compromising sleep quality”.*

Pain represents the principal symptom in OA. Nocturnal pain constitutes a notably significant aspect of the pain spectrum in OA. The occurrence of disruptive night pain is commonly employed by surgeons as a criterion in recommending TKR. Nocturnal pain constitutes a complex issue, affecting both sleep onset and maintenance. This pain intensifies based on the activities individuals engage in during the day, frequently encompassing routine tasks such as cooking, walking, shopping, participating in sports, or attending work. Sleep disturbances in individuals with osteoarthritis are correlated with heightened pain, increased fatigue, disability, and a propensity for depressed or anxious moods [20].

*“At night I don’t have the chance to get as comfortable in bed as I like.”*

(M, 72 years)

*“I sleep a maximum of five hours and I put a pad between my legs to have a good position and for pain.”*

(F, 70 years)

*“I sleep at night, but I always have pain in my knees, to get up I attach myself to the back of the bed otherwise I cannot.”*

(F, 70 years)

*“I wake up in pain depending on how much I have strained it during the day”.*

(F, 76 years)

## 4. Discussion

The primary objective of this study was to explore the preoperative experiences encountered by patients undergoing TKR surgery.

Regarding the first theme “The existence of pain impedes the capacity to participate in daily life activities”, the literature reveals the positive impact of TKR on health-related quality of life. Marked enhancements are noted in the domains of mobility, discomfort, and symptoms, alongside improvements in the domains of distress, daily activities, and vitality [21,22]. Clinical OA demonstrates a robust correlation with the capacity to perform activities of daily living (ADLs) in older adults. This relationship should be considered during clinical consultations when attending to patients with OA. Numerous patients conveyed a perpetual awareness of pain, depicting it as a constant presence that never subsides. Their narratives underscored the pivotal role that pain plays in their lives, illustrating its deep integration into their existence [23]. The significance of proficiently addressing pain, along with the provision of accurate information and support, has been substantiated to enhance confidence in both early discharge programs and postoperative management [24]. Persistent pain, a multifaceted and personalized phenomenon, constitutes the predominant symptom of osteoarthritis and can lead to diminished health quality and increased disability [25].

Within the domain of orthopedic patients, the assessment of pain commonly relies on Visual Analog Scales (VASs). Nevertheless, these scales exhibit multiple limitations as they neglect the nuanced nature of an individual’s pain experience [20].

Individuals afflicted with knee osteoarthritis harbor distinctive perspectives regarding their ailment and its treatment. They attribute paramount significance to pain within the framework of their lived experiences and recognize various factors influencing the intensity of pain perception [23]. The perspectives held by individuals concerning chronic pain exert a substantial influence on their attitudes and behaviors related to pain management. Frequently, there exists ambiguity regarding the etiology of and variability in chronic pain. In the absence of adequate information and guidance from healthcare providers, individuals may experience a deficit in understanding the appropriate measures to undertake. Consequently, they may resort to a strategy of avoiding physical activity to mitigate the perceived risk of exacerbating their condition [26,27].

Regarding the second theme “TKR induced fears and uncertainties regarding the progression of the disease”, from the literature, it is evident that patients commonly experience a pervasive sense of uncertainty regarding the surgery; a prevalent belief among patients is that undergoing surgery represents an inevitable juncture with no possibility of reversal [28]. Individuals experience confusion regarding the etiology of their pain and are perplexed by its unpredictable and variable nature. In the absence of sufficient information and guidance from healthcare professionals, individuals lack clarity on appropriate actions, leading to the avoidance of activities due to the fear of potential harm [26,29]. Gaining further insights into patients’ lived experiences with osteoarthritis (OA) and their expectations concerning knee arthroplasty is crucial. This exploration enhances our understanding of the impact of OA on their lives and provides valuable insights into their coping mechanisms with the disease. As written in the article by Josefina Nyvang et al., the need to limit physical activity leads to a widespread feeling of loss, as individuals are forced to let go of a fundamental element of their usual life. Social connections are impacted as they have to turn down certain activities, contributing to an overall sense of a restricted life with reduced spontaneity. A prevalent longing exists to restore past levels of physical activity, but the compromised state of the knee poses a barrier, hindering the fulfillment of these aspirations [30,31]. Anticipations regarding the surgical outcome are closely linked to what patients perceive as losses during the progression of the disease, such as diminished physical activity and decreased awareness of knee function [30].

Individuals diagnosed with osteoarthritis (OA) frequently encounter intensified nocturnal pain, leading to a decline in sleep quality. Research indicates a correlation between the reduction in sleep quality and the radiographic severity of the disease. Night pain constitutes a noteworthy aspect of the pain experience in hip OA [20]. In this study, the assessment of the mentioned nocturnal pain involves straightforward questions like “How do you sleep?” or “How is the quality of your sleep?”.

Regarding the third theme “Severe nighttime pain compromising sleep quality”, the literature documents that the association between sleep and pain is influenced by depressive symptoms, with sleep interacting with pain to magnify depression in individuals experiencing high levels of pain. Initial sleep disturbances predict elevated levels of depression and disability at follow-up, although not pain [32]. In individuals experiencing chronic osteoarthritis pain, the reduction in pain is linked to a decrease in sleep disturbances associated with pain [33]. The findings from certain studies suggest that an enhanced preoperative sleep quality may positively impact the pain threshold, leading to reduced pain scores and decreased consumption of analgesics [34,35]. The patients articulated experiences of both physical and mental fatigue attributed to pain and challenges in sleeping, indicative of a state of exhaustion [30].

Limitations inherent in this study may arise from the extrapolation of the results. Since this study was conducted in Italy with a group of participants consisting exclusively of Italians, variations could occur in countries with different cultures and customs. Therefore, future investigations should examine potential alterations in patients’ experiences after total knee arthroplasty, even in another context, to obtain a more comprehensive perspective. This study could be useful for the formulation of targeted educational and therapeutic strategies to improve care. The predominant inclusion of female participants in the sample could be considered a study limitation. However, recent advancements in gender medicine offer intriguing insights that may inform the design of future studies and facilitate comparative analyses with the male gender.

## 5. Conclusions

This study aims to shed light on the experiences of patients awaiting TKR. The present study reveals that patients commonly express a heightened sense of anxiety, particularly regarding the possibility of facing mobility challenges post-surgery. A major contributing factor to these concerns is the pervasive pain that adversely impacts their daily activities, persisting both day and night and thereby significantly compromising their overall quality of life. These findings are instrumental in advancing our understanding of the nuanced emotional landscape that patients navigate during the preoperative phase of TKR. Importantly, they emphasize the critical role of addressing specific emotional and informational needs. To enhance preoperative education and rehabilitation programs, it is imperative to acknowledge and tailor interventions to alleviate the apprehension associated with potential postoperative mobility issues and persistent pain. By doing so, this study suggests that healthcare professionals can contribute to a more comprehensive approach to patient care, ultimately improving the overall recovery and well-being of patients in the postoperative period.

## Figures and Tables

**Table 1:** Characteristics of participants.

Patients’ Characteristics	N (%)
Age	
70–75	5 (55.5%)
75–80	2 (33.3%)
>80	1 (11.1%)
Gender	
Male	1 (11.1%)
Female	8 (88.8%)

## Data Availability

The datasets used and/or analyzed during the current study are available from the corresponding author upon reasonable request.
